# Pathological (late) fractures of the mandibular angle after lower third molar removal: a case series

**DOI:** 10.1186/1752-1947-7-121

**Published:** 2013-04-30

**Authors:** Tommaso Cutilli, Theodora Bourelaki, Secondo Scarsella, Desiderio Di Fabio, Emanuele Pontecorvi, Pasqualino Cargini, Luis Junquera

**Affiliations:** 1 Department of Life, Health & Environmental Sciences, Maxillofacial Surgery Operative Unit, University of L’Aquila, via della Comunità Europea, 13, 67100, L’Aquila, Italy; 2 School of Dentistry, Oral and Maxillofacial Surgery, University of Oviedo, Oviedo, Spain

**Keywords:** Late mandibular angle fractures, Lower third molar removal, Early masticatory loading

## Abstract

**Introduction:**

Pathological (late) fracture of the mandibular angle after third molar surgery is very rare (0.005% of third molar removals). There are 94 cases reported in the literature; cases associated with osseous pathologies such as osteomyelitis or any local and systemic diseases that may compromise mandibular bone strength have not been included. We describe three new cases of pathological (late) fracture of the mandibular angle after third molar surgery.

**Case presentations:**

The first patient was a 27-year-old Caucasian man who had undergone surgical removal of a 3.8, mesioangular variety, class II-C third molar 20 days before admission to our clinic. The fracture of his left mandibular angle, complete and composed, occurred during chewing. The second patient was a 32-year-old Caucasian man. He had undergone surgical removal of a 3.8, mesioangular variety, class II-B third molar 22 days before his admission. The fracture, which occurred during mastication, was studied by computed tomography that showed reparative tissue in the fracture site. The third patient was a 36-year-old Caucasian man who had undergone surgical removal of a 3.8, vertical variety, class II-C third molar 25 days before the observation. In this case the fracture of his mandibular angle was oblique (unfavorable), complete and composed. The fracture had occurred during chewing. We studied the fracture by optical projection tomography and computed tomography.

All of the surgical removals of the 3.8 third molars, performed by the patients’ dentists who had more than 10 years of experience, were difficult. We treated the fractures with open surgical reduction, internal fixation by titanium miniplates and intermaxillary elastic fixation removed after 6 weeks.

**Conclusions:**

The literature indicates that the risk of pathological (late) fracture of the mandibular angle after third molar surgery for total inclusions (class II-III, type C) is twice that of partial inclusions due to the necessity of ostectomies more generous than those for partial inclusions. Other important factors are the anatomy of the teeth and the features of the teeth roots. These fractures predominantly occur in patients who are older than 25 years. The highest incidence (67.8% of cases) is found in the second and third week postsurgery. We emphasize that before the third molar surgery it is extremely important to always provide adequate instructions to the patient in order to avoid early masticatory loads and prevent this rare event.

## Introduction

The incidence of pathological (late) fracture of the mandibular angle after lower third molar (M3) surgical removal is approximately 0.005% [1-11].

The term “late” does not indicate that a fracture occurred at the conclusion of an operation of surgical avulsion of a lower M3 (in this case it would be an “immediate” complication), but in the period that begins when the patient has been discharged. So Perry and Goldberg [8] consider a late fracture to be indirectly related to the intervention and not a complication such as an “immediate” fracture which would be a direct consequence of the surgical procedure.

These iatrogenic fractures (in 0.0046% to 0.0075% of M3 removals) can, in fact, be avoided and prevented with accurate preoperative diagnostic study and the use of adequate surgical technique [12,13]; rhizotomy or section of the dental crown minimize the extent of bone removal and the force to be applied on the bone by the instrumentation [14]. The experience of the surgeon is very important [15].

The aim of the present paper is to describe three new cases of late fractures of the mandibular angle following lower M3 surgical removal and to debate the predisposing and related risk factors for this event.

## Case presentations

### Case 1

A 27-year-old Caucasian man was hospitalized to our clinic with swelling and pain in his left mandibular region.

**Figure 1:**
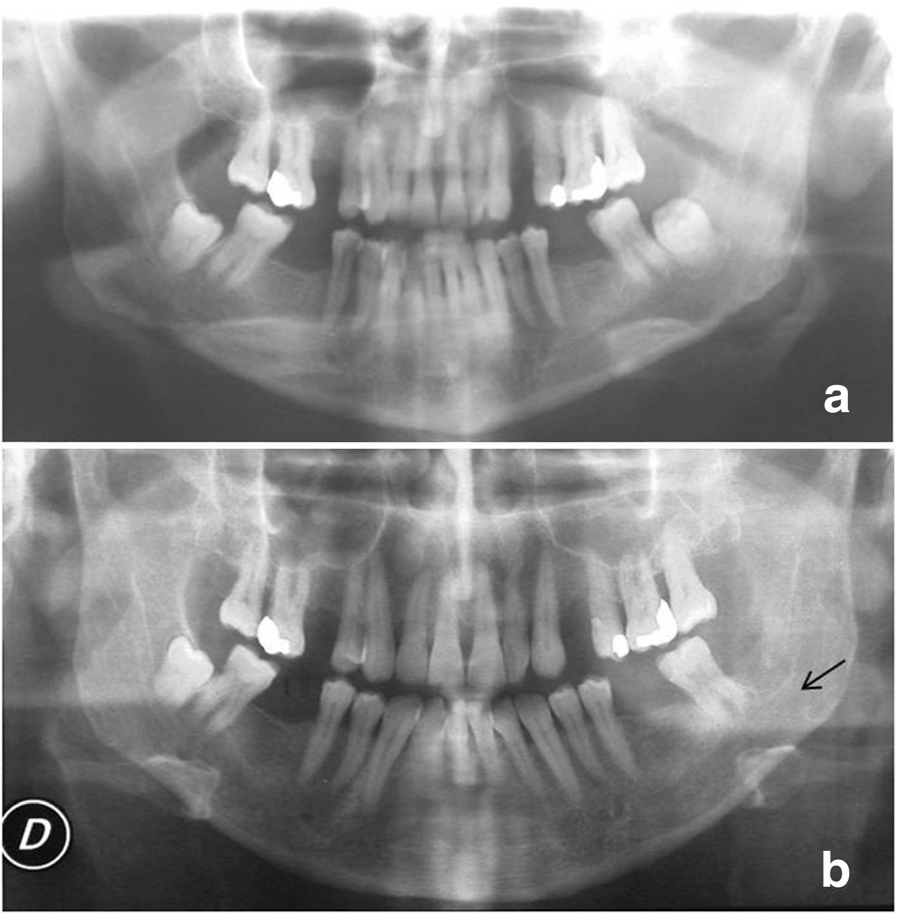
**(a) Panoramic radiography before 3.8 surgical removal.** Tooth appears with mesiodistal inclination and is class II-C. (**b**) Dental X-Ray 20 days after surgery shows the presence of a thin compound fracture of the left mandibular angle (arrow).

The patient had undergone a difficult surgery 20 days earlier to remove his left lower M3 (third molar) (3.8), mesioangular variety, class II-C (Figure 1a), under local anesthesia, performed by his own dentist. The postoperative course was regular and the phenomenon of postoperative edema was resolved. A few hours before his hospitalization, during masticatory movements, he felt a distinct cracking noise and pain in his left mandibular angle. Typical clinical signs and symptoms of mandibular angle fractures were present at his admission. A panoramic radiograph (Figure 1b) showed the fracture line in the site of extraction of his left inferior M3s, with evidence of its configuration and the position of the osseous fragments. The fracture appeared oblique, unfavorable (mesiodistal orientation), parallel to the long axis of the alveolar site, without dislocation of the fragments. The fracture was treated with open surgical reduction, internal fixation (IF) by miniplates and intermaxillary fixation (IMF) in normal occlusion using elastic bands that were removed after 6 weeks.

### Case 2

**Figure 2:**
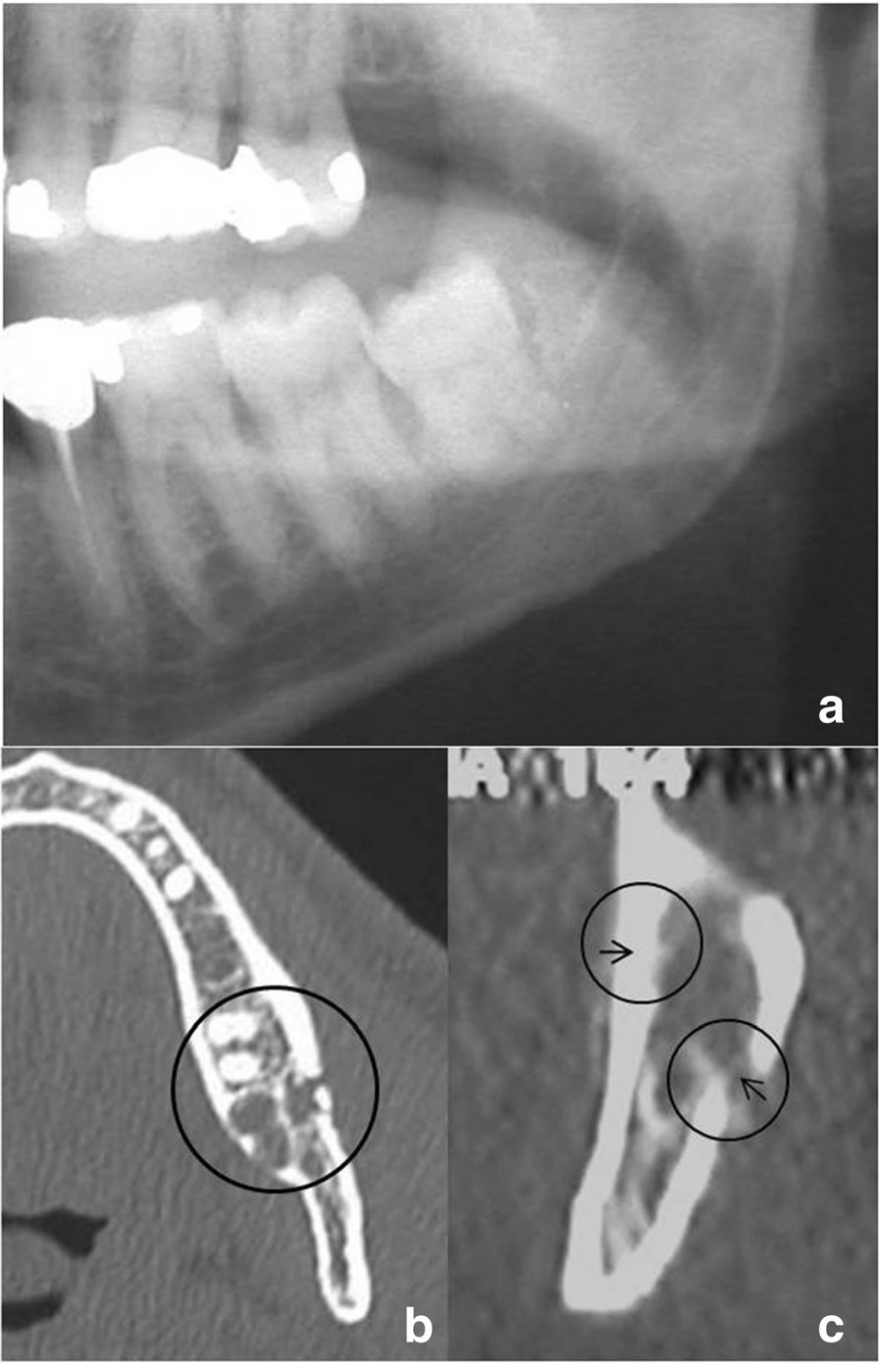
**(a) Panoramic radiography before 3.8 surgical removal.** Tooth appears with mesiodistal inclination and is class II-B. (**b**) Axial computed tomography (CT) scan shows fracture of the left mandibular angle that occurred 22 days postsurgery. The residual cavity of the extractive site with granulation tissue is visible (black circle). (**c**) The elements of Figure 2b as they appeared on a CT Dentascan reconstruction (black circles and arrows).

Our second patient was a 32-year-old Caucasian man who was referred to our clinic with swelling and pain in his left mandibular angle region. This patient had also undergone the surgical removal of a 3.8, mesioangular variety, class II-B third molar (Figure 2a). The operation, performed by his dentist under local anesthesia 22 days before his hospitalization, was very difficult and caused him considerable discomfort. Antibiotic and corticosteroid drugs were administered for 7 days. The postoperative phenomena were resolved completely after 15 days. The fracture of his left mandibular angle occurred during mastication. In addition to optical projection tomography (OPT) (Figure 2a), despite the fracture being composed, we performed a computed tomography (CT) (Figure 2b,c). The CT scan showed a complete fracture of his left mandibular angle that corresponded to the surgical site with reparative granulation tissue and absence of inflammatory phenomena. The fracture was treated with open surgery in the manner of Case 1.

### Case 3

**Figure 3:**
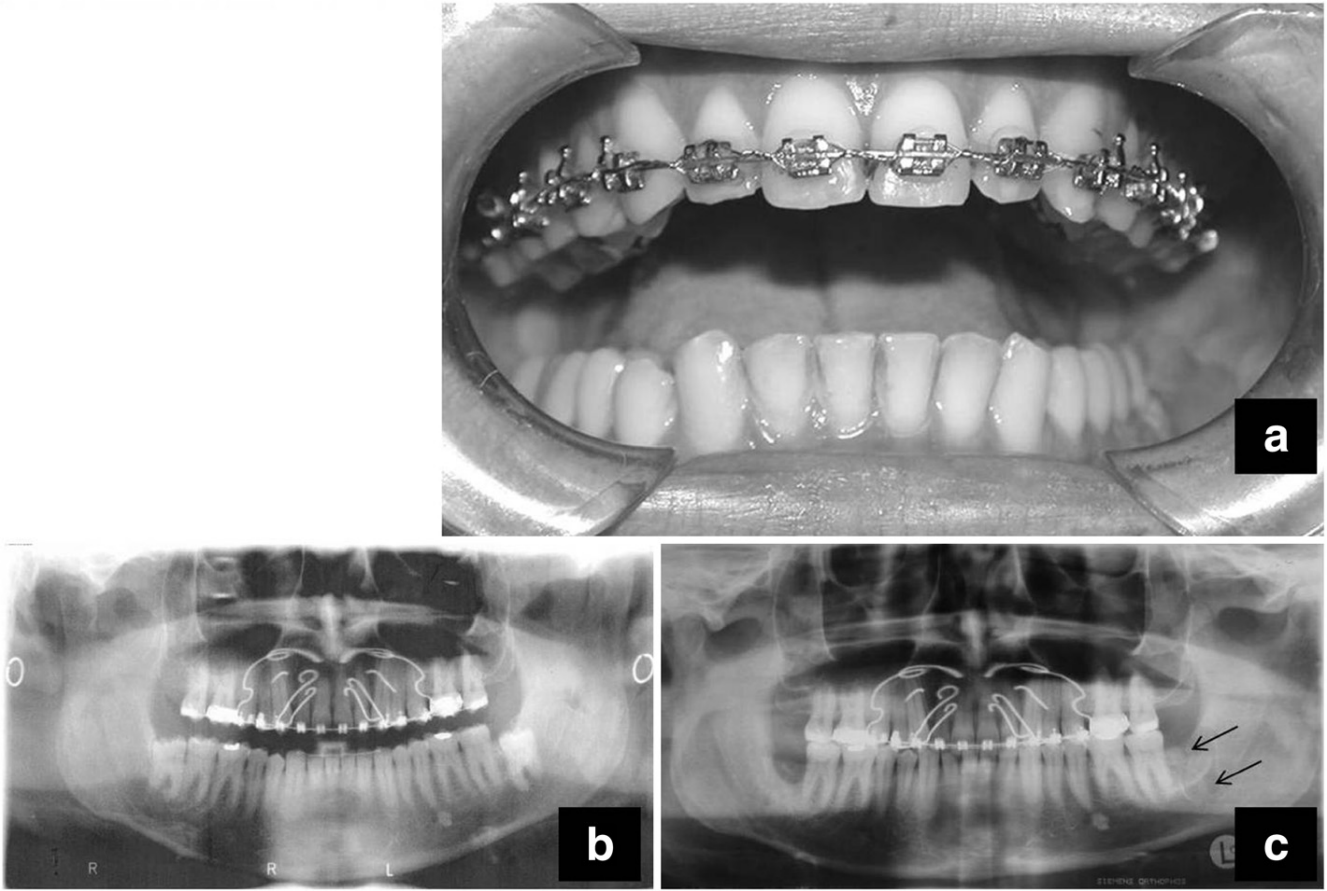
**(a) Reduced mouth opening.** (**b**) Dental X-Ray before 3.8 surgical removal. Tooth appears with vertical inclination and is class II-C. (**c**) Radiography 25 days postsurgery shows a compound fracture of the left mandibular angle (thin black arrows).

The third patient was a 36-year-old Caucasian man who was admitted to our clinic with swelling of his left mandibular angle region and severe pain. His mandibular movements were limited (Figure 3a,b). He had undergone surgical removal of a 3.8, vertical variety, class II-C third molar (Figure 3c), by his own dentist under local anesthesia, 25 days before the hospitalization. The mandibular fracture, which had occurred during mastication and was associated with pain, appeared linear on an OPT (Figure 3c).

**Figure 4:**
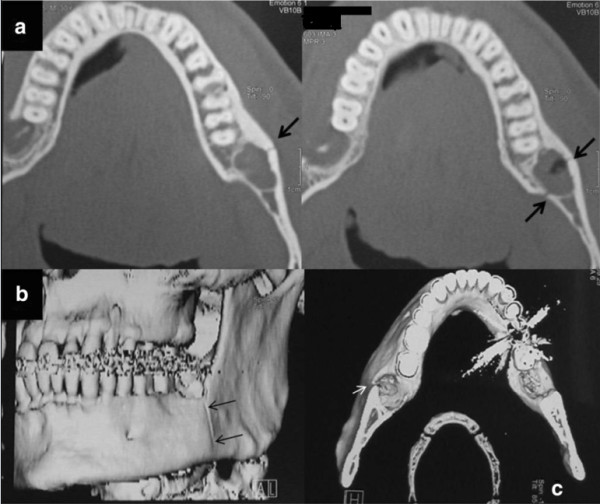
**(a) Axial computed tomography (CT) scans show the fracture of the left mandibular angle (black arrows) that involves the site of previous surgical removal of 3.8 where reparative tissue is still present.** (**b**) (**c**) The fracture as it appeared in a three-dimensional CT reconstruction (thin white arrow in C and black arrows in b).

**Figure 5:**
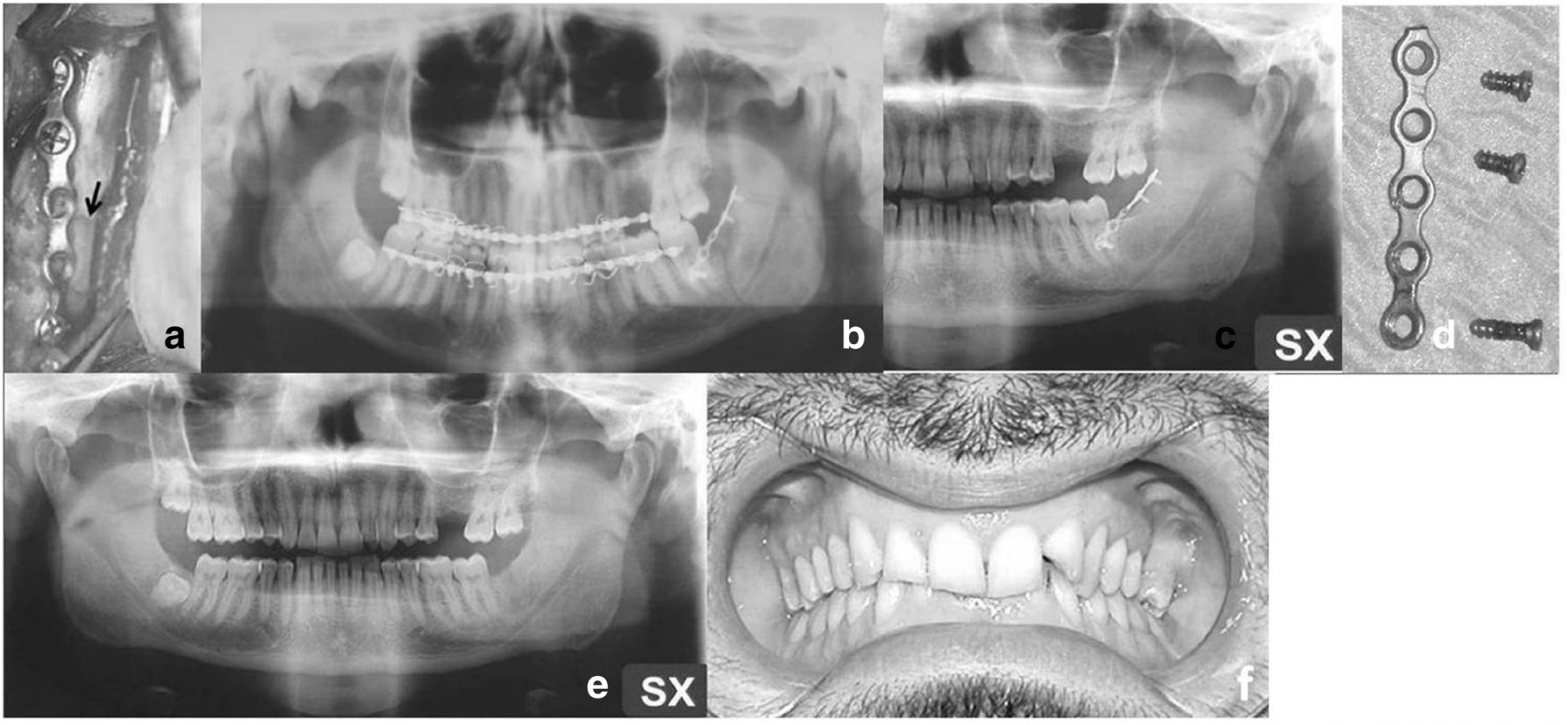
**(a) Surgical view of internal fixation (titanium miniplate) (arrow).** (**b**) Immediate postoperative Dental X-Ray shows a good reduction in the fracture. (**c**) Radiographic control executed 6 months postsurgery shows a good osseous repair. (**d**) The miniplate and the screws removed 16 months after surgery. The follow-up 3 years postoperative. (**e**) OPT shows good osseous restoration. (**f**) Good occlusal relationship.

A clinical examination detected a pathological motility of the left mandibular angle, so the diagnostic study was completed by CT (Figure 4a) and three-dimensional reconstructions (Figure 4b,c). These showed a complete fracture in correspondence to the site of 3.8 removal where there was reparative tissue. The patient underwent surgical treatment with IF by titanium miniplate (Figure 5a). Immediate postoperative radiographic control was executed (Figure 5b). A new radiographic control (Figure 5c) was executed 6 months postsurgery. We removed the titanium miniplate and the screws 16 months postsurgery (Figure 5d). The follow-up performed 3 years later showed a good radiological aspect of the left mandibular angle (Figure 5e) and of the occlusion (Figure 5f). The functional restoration of mandibular movements was also satisfactory.

## Discussion

Before their operations the three patients did not have mandibular pathologies or lesions or systemic disease that would compromise osseous structural integrity. They reported that they had undergone very difficult surgery to remove left mandibular M3s (third molars) (under local anesthesia, which had been performed by three different dentists who had more than 10 years of professional experience.

A distinct cracking noise, swelling in the mandibular angle region and pain were reported by the patients during chewing approximately 3 weeks after surgery.

At admission to our clinic, they presented with clinical signs and symptoms typical of mandibular angle fractures: swelling, occlusal alteration, pain, and functional reduction in opening their mouth.

Panoramic radiographs showed the fracture lines in the sites of extraction of the left inferior M3s, with evidence of their configuration and the position of the osseous fragments. All fractures were oblique, unfavorable (mesiodistal orientation), parallel to the long axis of the tooth removed, and without dislocation of the fragments. In two cases a CT study was also utilized. Panoramic radiographs executed before the M3 surgery were also available to evaluate radiological parameters such as tooth spatial position (dental angle), crown position and degree of impaction, according to Winter’s and Pell and Gregory’s classifications. The analysis of impacted teeth before surgery (Table 1) showed, respectively: mesioangular variety, class II-C; mesioangular variety, class II-B; and vertical variety, class II-C.

**Table 1:** Features of impacted teeth

**Patient**	**Tooth**	**Tooth position**	**Position of crown (class)**	**Degree of impaction (class)**	**Score of difficulty**
1	3.8	Mesioangular	II	C	7
2	3.8	Mesioangular	II	B	6
3	3.8	Vertical	II	C	8

We have treated the fractures with open surgical reduction, IF by titanium miniplates and IMF in normal occlusion using elastic bands, removed after 6 weeks.

The patients were recommended a soft diet for another 4 weeks. Panoramic radiographs, taken after 4 days, 3 and 6 months, 1 and 3 years postsurgery, revealed good evolution of osseous repair and complete structural recovery. Patients also showed a good functional recovery of mandibular movements and mastication. In one case (Case 3), on request of the patient the titanium miniplate was removed 16 months after surgery.

The removal of the inferior wisdom teeth is one of the most common procedures for oral and maxillofacial surgeons. Common complications of this procedure include alveolar osteitis (dry socket), secondary infection, neurological injuries, and hemorrhage. Iatrogenic damage or luxation of the second molar and locked trisma are less common complications. Frequent postoperative events are edema and swelling of the soft tissues, and pain. Excessive force for the mobilization of the impacted tooth can cause an incomplete or a complete iatrogenic intraoperative mandibular fracture, a rare but severe complication [13].

Pathological (late) mandibular fracture after M3 surgery is more uncommon but it is a major event, sometimes complicated. There are 94 cases reported in the literature (Table 2); cases associated with osseous pathologies such as osteomyelitis or any local and systemic diseases that may compromise mandibular bone strength have not been included [15].

**Table 2:** Cases of late fractures of mandibular angle after lower third molar surgery reported in the literature

**Author(s)**	**Year**	**Number of cases**
Harnisch [1]	1971	3
Haunfelder and Tetsch [2]	1972	4
Einrauch *et al*. [3]	1980	4
De Silva [4]	1984	1
Litwan and Goetzfried [5]	1987	4
Haertel *et al*. [6]	1988	4
Iizuka *et al*. [7]	1997	13
Perry and Goldberg [8]	2000	28
Krimmel and Reinert [9]	2000	6
Libersa *et al*. [10]	2002	10
Wagner *et al*. [11]	2005	17

Some elements that represent predisposing factors, related factors and risk are reported below. Certainly the excessive weakening of the mandibular angle, compared with the bone condition after routine M3 surgery, plays a decisive role. The following factors should be considered by the surgeon in the pre-operative time:

*Dental mass and relative volume of impacted tooth.* In our clinical experience there are cases in which an impacted tooth occupies a mandibular space of more than 50%.

*Type and class of tooth bone inclusion.* The risk for total inclusions (class II-III, type C) in which the M3 occupies a greater volume of bone and its removal requires ostectomies more generous than those for partial inclusions is twice that of partial inclusions. This implies a significant weakening of mandibular bone structure.

*Age of patients.* Osborne *et al*. [16] reported that 68% of patients included in his study were less than 25 years; Sisk *et al*. [15] and Goldberg *et al*. [17] indicate an average age, respectively, of 19 and 19.3 years. The incidence of complications of lower M3 surgical removal increases significantly after the third decade of life, in relation to the lower elasticity and compliance of the bone structure, and the frequent ankylosis of the impacted tooth; this makes the surgical procedure more difficult. In the cases reported by Harnisch [1], Iizuka *et al*. [7] and Wagner *et al*. [11], the average age at which surgery of M3 was performed was 49 ± 3 years and for these authors there is an increase in the incidence of fractures and their possible risk, especially in males with full dentition, for those patients older than 40 years. By contrast, in some studies no difference in incidence between patients who are 40 years old or older and those under 40 years emerges [18].

*Side*. The late fractures are more frequent in the left side than in the right side (for example as in the 70% of the patients reported by Wagner *et al*.). Most surgeons are right-handed and their control of the left surgical area is difficult; they have a poor view of the impacted 3.8, which makes it difficult to calibrate the forces applied on the mandibular structure [11].

*Time event*. Reports of the time at which the highest incidence of fracturative event occurs in the postoperative period include: in the second and third week (67.8% of 28 cases), with an average of 13 days [8]; in the first, second and fourth weeks (64.7% of 17 cases), with an average of 19 ± 4 days [11]. In our three cases, the fractures occurred 20, 22 and 25 days respectively after surgery with an average of 22.3 days. However, the event usually occurs within the first 4 weeks postsurgery [8]. In this period, in fact, the effect of surgery decreases and the patients feel better and begin to chew more easily, but in the surgical site the granulation tissue is replaced by connective tissue and in some cases two-thirds of the osteoid and bone tissue does not appear before the 38^th^ day [17]. So, after surgical removal of M3, especially if an extensive ostectomy has been performed, the mandibular angle region is not able to bear the normal masticatory loads from the second to the fourth week.

*Gender*. Late fractures occur because patients restart the full activity of chewing too early in relation to the osseous structural conditions. Men are more affected because they generally have greater muscular strength than women and they produce higher peak levels of biting forces than women [19].

All these aspects are usually not considered because of the low frequency of late mandibular angle fractures. Accordingly, after M3 surgical removal, surgeons do not give patients appropriate instructions on eating behavior. The regime of a soft diet should be extended beyond the end of the phenomena related to the intervention [11].

## Conclusions

Pathological (late) fractures of the mandibular angle after lower M3 surgical removal are an infrequent event [1-11]. The real percentage of late fractures is more difficult to define.

The preexistence of pathological bone alterations that cause a weakening of the mandibular structure such as periodontal disease, recurrent pericoronitis, osteoporosis, osteitis, and osteolytic lesions, that are more frequently present in patients who are 40 years or older, should also be considered.

The degree of tooth impaction and the ratio of tooth space to mandibular area are factors that indicate whether it is important to make an accurate preoperative diagnosis, obtained by panoramic radiography, but especially by CT Dentascan (CTD) or Cone Beam CT (CBCT), to avoid pathological (late) fractures of the mandibular angle. In fact, these advanced diagnostic methods show accurately in all planes of space, with axial scans and tridimensional reconstructions, the dental anatomy, the position of the impacted tooth and the ratio of tooth volume to mandibular angle volume. We utilize CTD or CBCT with all patients who are candidates for surgery of lower M3. In fact, it is also very important to always evaluate the relationship between lower M3 with the mandibular canal, regarding possible injuries of the alveolar nerve that could expose the surgeon to clinical and legal problems.

In conclusion, in cases of lower M3 surgical removal, it is appropriate that the surgeon:

a) **before surgery**, makes a proper assessment of the case and explains to the patient that, besides the usual complications, there is the possibility of a pathological (late) fracture of the mandibular angle that could occur during chewing;

b) **during surgery**, uses proper instrumentation, pays special attention to the procedure for 3.8 removal because it is usually more difficult than 4.8 surgery, performs a surgery that is conservative as possible, and does not exercise excessive force on the bone;

c) **after surgery** (especially after a difficult operation with severe bone removal and impairment), explains to the patient the importance of an appropriate food style for an adequate period of time (for at least 4 to 5 weeks).

The possibility that the patient will not follow these recommendations must often be considered a determinant factor for a fracturative event. Surgeons should also explain to the patient how to recognize the symptoms of a late fracture and the need to treat it surgically.

## Consent

Written informed consent was obtained from the patients for publication of this manuscript and any accompanying images. Copies of the written consents are available for review by the Editor-in-Chief of this journal.

## Competing interests

The authors declare that they have no competing interests.

## Authors’ contributions

TC designed and coordinated the work and was the main surgeon who collected the data and corrected the manuscript. TB analyzed and interpreted the patient data regarding the anamnesis. DDF was a major contributor in writing the manuscript. SS supervised the translation of the manuscript. EP wrote the bibliography. PC prepared the images and revised the article. LJ supervised the discussion and the conclusions. All authors read and approved the final manuscript.
